# SGLT2 Inhibitor–Associated Euglycemic Diabetic Ketoacidosis (EDKA) in Hematologic Malignancy Patients: A Case Series

**DOI:** 10.1155/ah/7244013

**Published:** 2025-10-08

**Authors:** Andrew Artz, Tiffany Nguyen, Raynald Samoa, Hoim Kim, Wyndie Tse, Rami Jin, Svetlana Goutnik

**Affiliations:** City of Hope, Duarte, California, USA

**Keywords:** euglycemic DKA, hematology, SGLT2 inhibitor

## Abstract

Sodium-glucose Cotransporter 2 inhibitors (SGLT2i) are widely used and effective pharmacotherapeutic options that are first-line therapy for Type 2 diabetes mellitus (T2DM) and congestive heart failure. A major adverse effect of SGLT2i usage is diabetic ketoacidosis (DKA). SGLT2i-induced DKA commonly presents as euglycemic DKA (EDKA). The safety of empagliflozin in cancer patients is not well established. High intensity treatment of hematologic malignancy poses a unique set of risk factors for EDKA. Four cases of empagliflozin-associated EDKA in hematologic malignancy patients were identified through pharmacy adverse event reporting at a cancer research hospital. All patients were euglycemic except one patient who required CRS/ICANS treatment with dexamethasone, causing steroid-induced hyperglycemia. All four patients had weight loss due to reasons including, but not limited to, reduced oral intake, diarrhea, nausea, and pain. Infections and/or neutropenic fever were also commonalities throughout the four patients. In all patients, DKA contributed to iatrogenic ICU admissions and prolonged hospital stays. SGLT2i use in hematologic malignancy patients may increase the risk of DKA due to high risk of anorexia, weight loss, and infections, all highly associated with intensive treatment that can disrupt the availability and sensitivity to insulin. In patients receiving SGLT2i, clinicians should be aware of risk factors for DKA as well as potential euglycemic presentation to ensure close clinical and laboratory monitoring to facilitate rapid diagnosis and treatment of DKA.

## 1. Background

Sodium-glucose Cotransporter 2 inhibitors (SGLT2i) are widely used and effective pharmacotherapeutic options that are first-line therapy for the treatment of Type 2 diabetes mellitus (T2DM) [1], congestive heart failure [2], reduction in cardiovascular mortality, and delay in progression of chronic kidney disease in patients with Type 2 diabetes mellitus and established cardiovascular disease.

A major adverse effect of SGLT2i usage is diabetic ketoacidosis (DKA), a serious acute complication resulting in metabolic acidosis and increased total body ketone concentration [3]. The diagnostic criteria for classic DKA is a plasma glucose ≥ 200 mg/dL, arterial pH 7.25–7.3, serum bicarbonate 15–18 meq/L, and *β*-hydroxybutyrate 3.0–6.0 mmol/L [3]. SGLT2i-induced DKA commonly presents as euglycemic DKA (EDKA), with plasma blood glucose < 250 mg/dL [4], since the glucosuria caused by these agents can mask hyperglycemia. The initial glucose presentation is the key difference in diagnostic criteria between DKA and EDKA, as can be seen in Table 1.

The absence of typical characteristic hyperglycemia may result in diagnostic challenges of EDKA which can delay treatment and increase the risk of a negative outcome. DKA occurs in situations where not enough insulin is being produced [5], thus is more common in Type I diabetes, and requires insulin therapy, usually provided via an intravenous continuous infusion. Patients on SGLT2i for other indications, such as heart failure and T2DM, are at risk due to high catabolic stress during periods of acute illness, such as trauma, surgery, or infections. DKA can occur due to (1) insulinopenia, (2) elevation of counter regulatory stress hormones, and (3) increase in free fatty acids [6]. After multiple postmarketing reports of EDKA in patients on SGLT2i, the US Food and Drug Administration (FDA) released a recommendation against the use of these agents in patients with Type I diabetes mellitus. Due to the difficulty in diagnosing EDKA, in May 2015, the FDA released a statement that recommended providers assess for signs and symptoms of ketoacidosis in patients taking SGLT2i, such as nausea, vomiting, abdominal pain, fatigue, or shortness of breath, instead of solely relying on the presence of elevated glucose levels [7].

The original study of empagliflozin (Jardiance) for the treatment of T2DM [5] excluded patients with a past medical history of cancer (except for basal cell carcinoma) and/or treatment for cancer within the last 5 years. Thus, the safety of empagliflozin in cancer patients is not well established. There are few studies describing the incidence of DKA in solid tumor patients on SGLT2i and none in hematologic malignancy patients. One case series comprised of different solid tumors identified poor oral intake and infections as the primary risk factors for SGLT2i-associated DKA [9]. There are few reports published on EDKA in cancer patients, with one case report in pancreatic cancer identifying starvation as a key precipitating factor [10] and another case report in metastatic lung adenocarcinoma identifying chemotherapy as the trigger for DKA [11]. Both case reports point to early recognition of precipitating factors as a crucial point, due to the ability of SGLT2i agents to mask hyperglycemia. The manufacturer label for empagliflozin recommends monitoring for ketoacidosis and temporarily discontinuing empagliflozin in situations known to predispose to ketoacidosis, such as prolonged fasting due to acute illness or surgery [12].

High intensity treatment of hematologic malignancy, for example, stem cell transplant, high-intensity chemotherapy, and chimeric antigen receptor (CAR)–T-cell therapy, poses a unique set of risk factors for EDKA. Higher intensity treatment or conditioning before hematopoietic cell transplantation frequently causes diarrhea, mucositis, pain, infection, and other acute illnesses. Guidance on the benefits and risks of continuing SGLT2i during high intensity treatment is currently unavailable. We describe four cases of SGLT2i-associated DKA in hematologic malignancy patients receiving intensive treatment in order to identify risk factors that may predispose this patient population to the development of DKA.

## 2. Case Presentation

Four cases of empagliflozin-associated EDKA in hematologic malignancy patients were identified through pharmacy adverse event reporting from December 1, 2023, to April 30, 2024, at a cancer research hospital. The City of Hope institutional review board approved the study with a waiver of informed consent. EDKA risk factors, presentation, and outcomes are described in Table 2. Only Patient 2 had an elevated serum creatinine (1.21 mg/dL) at the time of EDKA presentation, and all patients had normal hepatic function (total bilirubin, alkaline phosphatase, aspartate transaminase [AST], and alanine aminotransferase ALT]). All four patients were male, with a mean age of 54 years (range 34–79 years). Three out of four patients had recent hematopoietic stem cell transplant (HCT) or CAR–T-cell therapy (lisocabtagene maraleucel).

All patients received SGLT2i for T2DM. All patients were sent to the intensive care unit (ICU) for treatment of DKA with an insulin drip, and inpatient SGLT2i was discontinued. Mean duration of insulin drip was 23 h (ranging 13–36). Mean length of ICU stay was 4 days (ranging 2–6). Additional complications, such as neutropenic fever and immune effector cell-associated neurotoxicity syndrome (ICANS), lengthened the ICU stay for some patients. Mean hospital length of stay was 13 days (ranging 5–24), due to post-transplant and CAR-T monitoring.

## 3. Discussion

All four patients had weight loss due to reasons including, but not limited to, reduced oral intake, diarrhea, nausea, and pain. Infections and/or neutropenic fever were also commonalities throughout the four patients. One patient had CRS/ICANS following CAR-T therapy that may be considered an acute illness that could in turn contribute to the development of DKA. In all patients, DKA contributed to iatrogenic ICU admissions and prolonged hospital stays.

**Figure 1:**
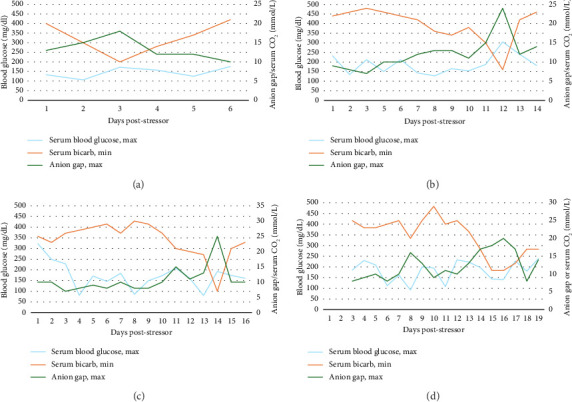
Daily laboratory assessment of DKA. (a) Patient #1. (b) Patient #2. (c) Patient #3. (d) Patient #4.

For patients receiving SGLT2i, evaluation of daily laboratory assessments beyond glucose alone may aid in quickly diagnosing DKA as euglycemia may delay diagnosis. All patients were euglycemic except Patient 2, although this patient required CRS/ICANS treatment with dexamethasone, likely causing steroid-induced hyperglycemia.  Figure 1 displays the daily laboratory abnormalities along the timeline of DKA development in all four patients. The day of EDKA diagnosis was marked by the drop in serum bicarbonate and spike in anion gap.

On the day of DKA diagnosis, the mean plasma glucose was 210 mg/dL (ranging 141–305), the mean anion gap was 22 (ranging 18–25), the mean serum bicarbonate was 9 meq/L (ranging 7–11), and the mean pH was 7.25 (ranging 7.21–7.32).

Monitoring serum blood glucose, therefore, should not be the only notable parameter while patients are on SGLT2i. pH and urine ketones are not routinely ordered. Therefore, daily monitoring of serum bicarbonate and anion gap is likely the easiest ways to monitor for emergence of DKA since they are included in routine bloodwork. Careful attention to these laboratory parameters will aid in swift diagnosis and treatment of DKA in hospitalized patients that are continued on SGLT2i. As a case series, the actual incidence or risk related to empagliflozin or other SGLT2i requires further research among hematologic malignancy patients. Moving forward, having a profile of patients at risk for EDKA such as weight loss, poor intake, and high intensity treatments would increase visibility of this condition. Additionally, hospital policies should be implemented to identify patients at risk and clearly display the steps toward decreasing the risk of EDKA to prevent iatrogenic ICU admissions, prolonged lengths of stay, and unnecessary patient harm.

## 4. Conclusions

SGLT2i use in hematologic malignancy patients may increase the risk of DKA due to high risk of anorexia, weight loss, and infections—conditions highly associated with intensive treatment that can disrupt the availability and sensitivity to insulin. In patients receiving SGLT2i, clinicians should be aware of risk factors for DKA as well as potential euglycemic presentation to ensure close clinical and laboratory monitoring to facilitate rapid diagnosis and treatment of DKA. SGLT2i containing evolving indications and benefits continue to expand utilization. This case series suggests policies on the use of SGLT2i in hematologic malignancy patients receiving high intensity therapy to guide appropriate monitoring for EDKA in the setting of ongoing SGLT2i therapy.

## Figures and Tables

**Table 1:** Diagnostic criteria for DKA per the ADA guidelines, with the main difference with EDKA being the lack of severely elevated glucose levels.

DKA diagnostic criteria	EDKA diagnostic criteria [4]
Glucose ≥ 200 mg/dL OR prior history of diabetes	Glucose < 250 mg/dL
β-Hydroxybutyrate ≥ 3.0 mmol/L OR urine ketone Strip 2+ or greater	
pH < 7.3 and/or bicarbonate < 18 mmol/L	

**Table 2:** EDKA risk factors, presentation, and outcomes.

Patient	Weight loss^†^	Infectious risk	Noninfectious risk	Malignancy	Treatment	Day post-HCT/CAR of hospital admission	Day post-HCT/CAR of EDKA presentation	Reason for hospital admission
1	4 kg (3.6%)	RSV	Abdominal pain Diarrhea	AML	MUD allogeneic HCT	D + 164	D + 164	Epigastric pain
2	4 kg (4.3%)	COVID-19	Altered mental status, ICANS Concurrent dexamethasone	B-cell lymphoma	CAR-T with cyclophosphamide and fludarabine conditioning	D + 1	D + 6	CRS grade 1
3	5.3 kg (8.9%)	Culture-negative neutropenic fever	Nausea, vomiting, diarrhea	B-cell lymphoma	Autologous HCT with BEAM conditioning	D + 7	D + 7	EDKA
4	6.1 kg (6.6%)	RSV *E. coli* bacteremia	Mucositis Diarrhea	ALL	MSD allogeneic HCT with palifermin/etoposide/FTBI conditioning	D − 4	D + 5	HCT

**Patient**	pH^∗^	Anion⁣gap^∗^	Blood⁣glucose^∗^**(mg/dL)**	**Empagliflozin dose**	**Other diabetic home medications**	**Duration of inpatient resumption of SGLT2i (days)**	**Length of ICU admission (days)**	**Total length of stay (days)**

1	7.2	15	106	25 mg daily	Metformin Insulin	2	2	6
2	7.21	24	305^∗∗^	10 mg daily	Dulaglutide Insulin	6	6	15
3	7.32	25	193	12.5 mg daily	Metformin Insulin	0^§^	2	5
4	7.25	20	141	25 mg daily	Pioglitazone Insulin	10	6	24

*Note:* BEAM, carmustine, etoposide, cytarabine, and melphalan; COVID-19, coronavirus; HCT, hematopoietic stem cell transplant.

Abbreviations: ALL, acute lymphoblastic leukemia; AML, acute myeloid leukemia; CAR, chimeric antigen receptor; CRS, cytokine release syndrome; EDKA, euglycemic diabetic ketoacidosis; MSD, matched sibling donor; MUD, mismatched unrelated donor; RSV, respiratory syncytial virus.

^†^Weight loss during the week prior to EDKA diagnosis.

^§^Patient readmitted after a 3-day admission for febrile neutropenia (culture-negative). He was not restarted on SGLT2i during current or previous admission but was noted to be taking SGLT2i at home in-between admissions.

^∗^Maximum blood glucose and anion gap, minimum serum bicarbonate and pH on the day of EDKA diagnosis.

^∗∗^Received dexamethasone for treatment of ICANS.

## Data Availability

The data that support the findings of this study are available from the corresponding author upon reasonable request. Due to privacy concerns and institutional guidelines, the data are not publicly available but may be shared upon request for research purposes.
